# The prevalence of stress during the COVID-19 pandemic: an umbrella review

**DOI:** 10.1186/s12889-025-24922-3

**Published:** 2025-11-18

**Authors:** Mostafa Amini-Rarani, Sepideh Rezaei, Saber Azami-Aghdash, Salman Bashzar, Shahideh Allahverdi, Mohammad Mohseni

**Affiliations:** 1 Social Determinants of Health Research Center, Isfahan University of Medical Sciences, Isfahan, Iran; 2 Student Research Committee, School of Management and Medical Information Sciences, Isfahan University of Medical Sciences, Isfahan, Iran; 3 Tabriz Health Services Management Research Center, Tabriz University of Medical Sciences, Tabriz, Iran; 4 Health Management and Economics Research Center, Isfahan University of Medical Sciences, Isfahan, Iran

**Keywords:** Stress, Prevalence, COVID-19, Pandemic, Meta-analysis

## Abstract

**Background:**

The COVID-19 pandemic has caused widespread mental health problems, with stress affecting a large porportion of the global population. To provide a comprehensive understanding of the need for post-pandemic mental health services, this umbrella review was conducted to accurately estimate the prevalence of stress during the COVID-19 pandemic.

**Methods:**

Web of Science, PubMed, Scopus, and Embase were searched for published meta-analyses using relevant keywords, such as prevalence, stress, COVID-19, and Meta-analysis up to January 1, 2025. Additional manual searches were performed in selected journals and through Google Scholar to identify further relevant articles. A random-effects model was used for the analyses. All analyses were conducted using STATA 17 software.

**Results:**

Of 3697 records screened, 45 meta-analyses were included. The pooled prevalence of stress was 41% [95% CI: 36–45] with high heterogeneity (I^2^: 93.22%). The highest prevalence was observed in patients (56% [49–63]) and health-care workers (45% [38–52]). The prevalence of stress was higher in females (40% [18–63]) compared with males (27% [3–50]). In terms of severity, the highest percentage was related to moderate 29% [7–50], mild 24% [6–41], and severe 13% [5–21].

**Conclusions:**

Stress was highly prevalent during the COVID-19 pandemic, particularly among patients, healthcare workers, pregnant womens, and students. Policy responses should prioritize funding, advocacy and system-level interventions to mitigate the mental health impact of pandemics and strengthen resilience in preparation for future public health crises.

## Background

Over the centuries, humans have confronted numerous infectious diseases that have caused millions of deaths, leading to the evolution of resistance against these illnesses [1]. Nowadays, both emerging and re-emerging infectious diseases remain significant challenges to human well-being [2]. For example, on March 11, 2020, the novel coronavirus disease (COVID-19) was declared a global pandemic. The uncontrollable and unpredictable nature of COVID-19 resulted in significant stress among the general population [3], which was further exacerbated by increased financial pressures and social isolation [4], as well as fear of infection for oneself or loved ones [5].

To mitigate the risk of exposure to COVID-19, social distancing measures were recommended and implemented. People from all walks of life were required to stay at home and maintain physical distance whenever they were outside [6, 7]. This intervention not only affected all ongoing activities but also had a profound negative impact on individuals’ mental health. Additionally, fear of infection, unavailability of effective treatment, mortality, economic losses, disruption of daily routines and continuous exposure to news were other factors that adversely affected mental health. Consequently, the COVID-19 crisis became an uncontrollable stressor [8].

The World Health Organization has warned that at least one-third of individuals exposed to a pandemic such as COVID-19 experience psychological manifestations. Evidence from studies demonstrates that anxiety, depression, and stress are prominent emotional responses to the COVID-19 epidemic, affecting approximately half of the population with varying levels of severity [9, 10]. In prior pandemics, including SARS, stress prevalence was reported to be as high as 68% [11] to 80% [12]. Importantly, follow-up assessments indicated that, one year after the outbreak, healthcare workers exhibited higher stress levels than the general population [13].

According to one study, 59% of men infected with COVID-19 exhibited normal stress levels, 12.3% moderate stress, 11.5% severe stress, and 9% very severe stress. In contrast, nearly half of the female participants (49.4%) had normal stress levels, 11.5% moderate stress, 12.6% severe stress, and 13.8% very severe stress [14].

In recent years, given the increasing and high rates of mental health disorders during the COVID-19 pandemic, as well as the critical importance of pandemics and their related statistics, numerous researchers have conducted systematic reviews and meta-analyses in this field. Systematic aggregation of primary study results and meta-analysis can provide a more comprehensive and accurate estimate of the current situation. Despite providing comprehensive and valuable information, some of these studies appear to have conflicting results. Moreover, the large number of such studies has posed challenges for decision-makers and administrators in selecting and utilizing their findings effectively. Furthermore, due to significant differences in the results of systematic reviews and variations in the scope of these studies—such as differences in age groups, occupations, geographic regions and other factors related to primary studies on disease prevalence, conducting systematic reviews and meta-analyses sometimes leads to fragmented data and statistics. This fragmentation may cause confusion among policymakers and decision-makers. Therefore, it appears that performing a meta-analysis of existing meta-analyses can substantially reduce these discrepancies and provide more comprehensive, accurate and reliable information (given the large combined sample size) for decision-makers, policymakers, healthcare providers and other stakeholders. For example, categorizing and analyzing common cases with larger sample sizes and aggregating their results can yield better and more credible outcomes. Hence, the present study aims to conduct a meta-meta-analysis of meta-analyses estimating the prevalence and severity of stress during the COVID-19 pandemic.

## Methods

This umbrella review designed and conducted in 2025 according to the Preferred Reporting Items for Systematic Reviews and Meta-Analyses (PRISMA) [15]. The Institutional Review Board and the Ethics Committee of Isfahan University of Medical Sciences approved this study (Ethics code: IR.MUI.NUREMA.REC.1404.056). In addition, the protocol for this review was registered with the PROSPERO (CRD42024500045).

### Search strategy

An expert librarian with substantial experience in the field designed and executed the search strategy. Two independent reviewers systematically searched major electronic databases-including Web of Science, PubMed, Scopus, and Embase-using relevant MeSH terms, covering literature published up to January 1, 2025. Additional manual searches were performed in selected journals and via Google Scholar to identify further pertinent articles. The search included the following keywords: “Mental health”, “Mental disorders”, “Stress”, “Psychological disorders”, “Psychological impact”, “Prevalence”, “Coronavirus”, “COVID-19”, “Pandemic”, “Meta-analysis” and other related keywords (Appendix 1-Search strategy). Reference lists of the included studies and hand-searching were also used to ensure all relevant literature was accessed.

### Inclusion and exclusion criteria

This review included all meta-analyses published in English globally that reported on the prevalence of stress during the COVID-19 period. Studies were excluded if they met any of the following criteria: 1) they were not meta-analyses, 2) they were narrative reviews, comprehensive reviews, editorials, communications, or brief reports, 3) they had not undergone peer review the, 4) the full text was unavailable or inaccessible, or they were rated as having low reporting quality, defined as scoring below 5 out of 11 on the reporting quality assessment tool.

### Study screening and selection

Search results were managed and duplicate entries were removed using Endnote X9. Two authors (SAA and MM) independently screened the titles and abstracts of the identified studies. Any disagreements between them were resolved through discussion with a third author (MAR). All stages of article selection and screening were performed independently by two researchers. Initially, titles were assessed and studies not aligning with the aims of the study were excluded. Subsequently, abstracts and full texts were evaluated to further exclude studies that met the exclusion criteria or were only weakly related to the research aims. Endnote X9 was utilized for organizing references, screening titles and abstracts, and detecting duplicates. The PRISMA 2020 flowchart was used to report the study selection and screening process.

### Quality assessment

Two independent reviewers assessed the reporting quality of all full-text articles using the AMSTAR tool (Assessment of Multiple Systematic Reviews) [16]. Each item on the tool was rated as “Yes” (scored as 1), “No,” “Cannot answer,” or “Not applicable” (each scored as 0). Articles were categorized based on their total score: 1–4 points indicated low quality, 5–8 points medium quality, and 9–11 points high quality. The final quality rating for each article was determined by agreement between the two reviewers. If disagreements arose, they were resolved by consensus or, if necessary, by consulting a third reviewer.

### Data extraction

Data extraction was carried out independently by two reviewers using a standardized extraction form. Extracted information included: sample size, number of studies included in each meta-analysis, total population reviewed, date of the last literature search, time interval between the start of the COVID-19 pandemic (December 15, 2019) and the last search date, prevalence rates of stress, severity, and quality score. Any differences in data extraction were resolved by consensus or by involving a third reviewer.

### Data synthesis

A random effects model was used for the meta-analysis, which allows for the generalization of findings by considering that the included studies represent a random sample from a broader population [17]. All analyses were performed using STATA version 17, and the results were visually summarized using forest plots. In these plots, the size of each square represents the sample size, while the lines extending from the squares indicate the confidence intervals (CI). Study heterogeneity was evaluated using the I² statistic, with values interpreted as follows: low (< 50%), moderate (50–74%), and high (>75%) heterogeneity [18]. Sensitivity analysis was conducted to explore the source of heterogeneity. Meta-regression was conducted to examine the relationship between the time elapsed from the onset of the COVID-19 pandemic (December 15, 2019) and the last search date (measured in weeks) in the included studies. Publication bias was assessed using funnel plots, as well as Begg’s and Egger’s tests. Additionally, a trim and fill analysis with a linear estimator was performed to further evaluate publication bias.

### Subgroup analyses

Subgroup analyses were performed according to population type (including general population, healthcare workers, patients, pregnant/postpartum women, students), severity of stress (mild, moderate, severe), and gender (male, female).

## Results

**Fig. 1:**
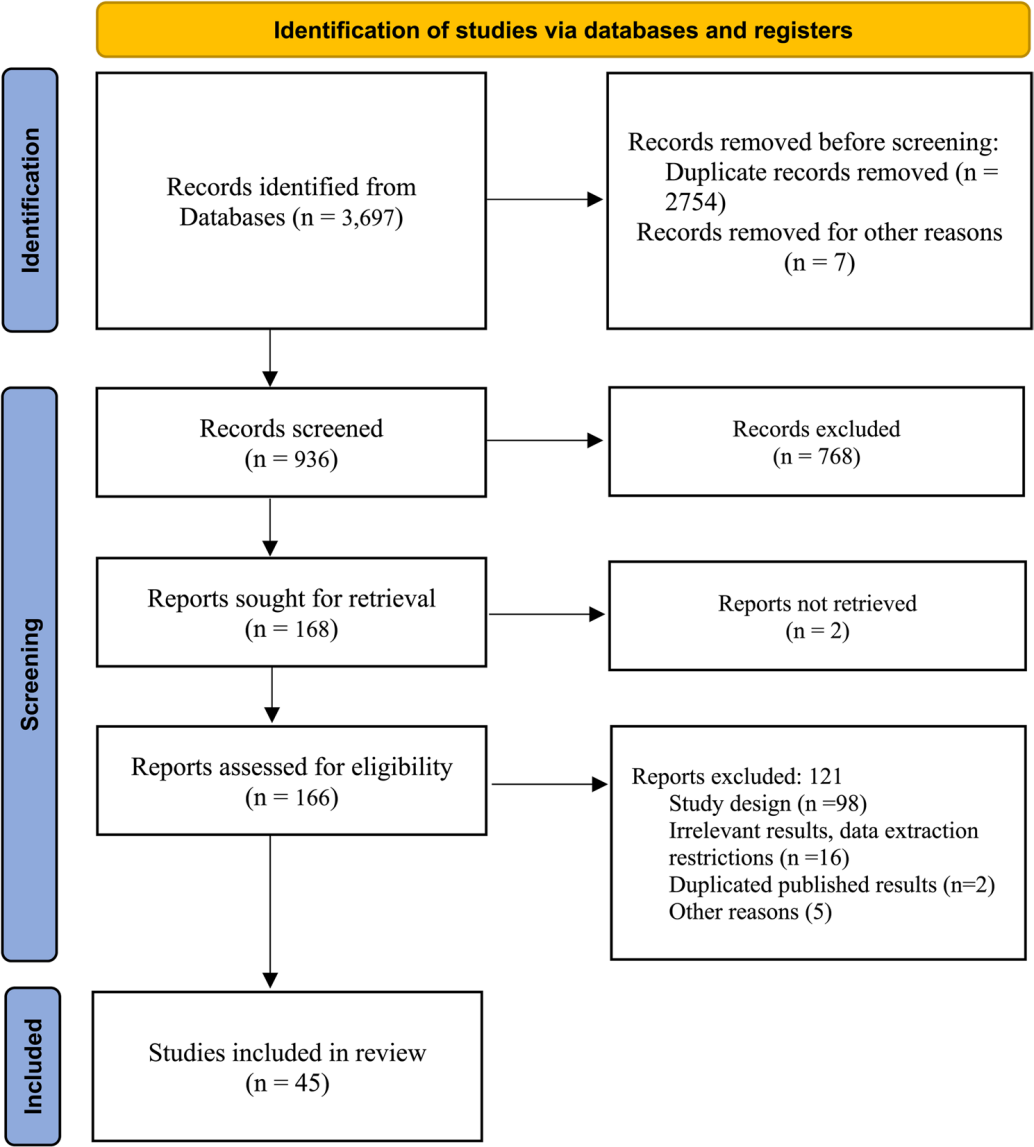
PRISMA flowchart

The systematic search identified 3,697 records from databases and other sources. After removing 2,761 duplicate records and studies that did not meet the inclusion criteria, 936 records were subjected to title and abstract screening, resulting in the exclusion of 768 studies. The full-text assessment of the remaining 166 articles led to the exclusion of 121 studies. Ultimately, 45 studies [19–63] met al.l eligibility criteria and were included in the meta-analysis (Fig. 1). The characteristics and results of the included studies are listed in Appendix 2.

The largest proportion of published articles was in 2021 (37.8%). The subsequent publication rates were distributed as follows: 2022 (22.2%), 2024 (20%), 2020 (8.9%), 2023 (6.7%) and 2025 (4.4%). Healthcare workers were the most frequently studied population group in all publications (60%), followed by the general population (17%), students (8.9%) and both pregnant/postpartum women and patients (6.7% each). The time frame for the stress assessment varied considerably, with measurement intervals ranging from 11 weeks to 215 weeks following the COVID-19 pandemic onset.

### Overall and subgroup prevalence

**Fig. 2:**
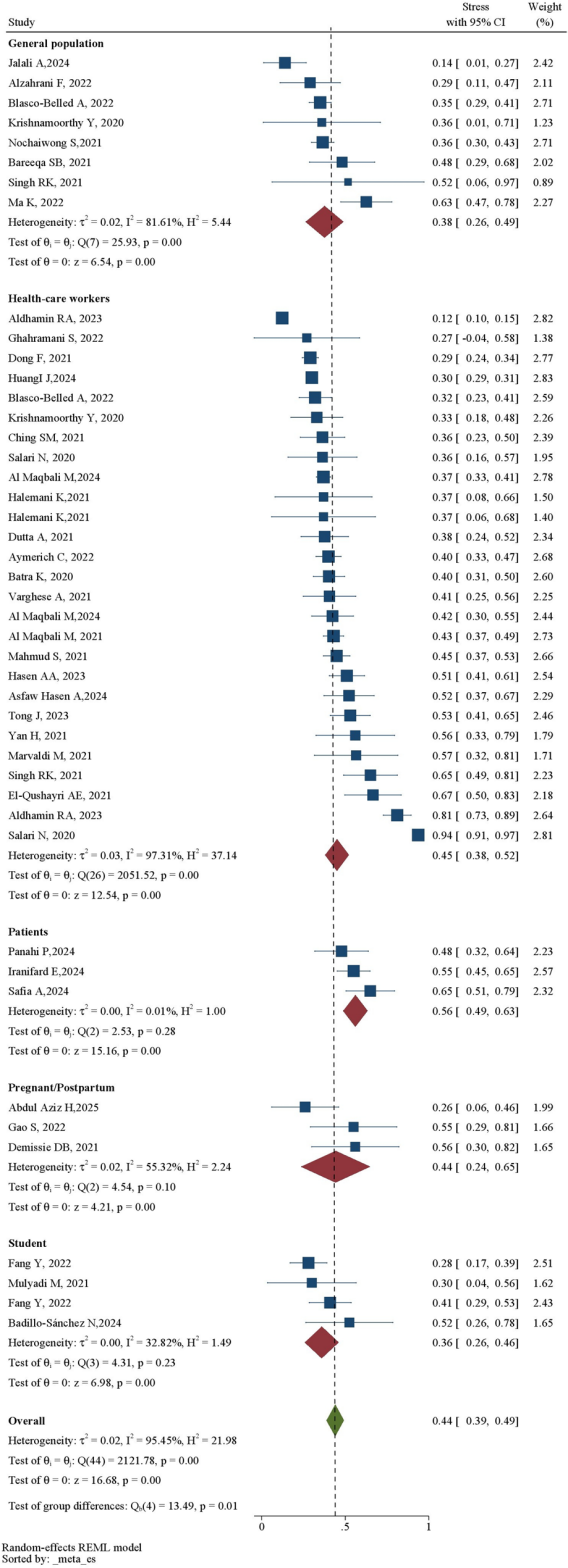
Prevalence of stress during the COVID-19 according to the subgroups

**Fig. 3:**
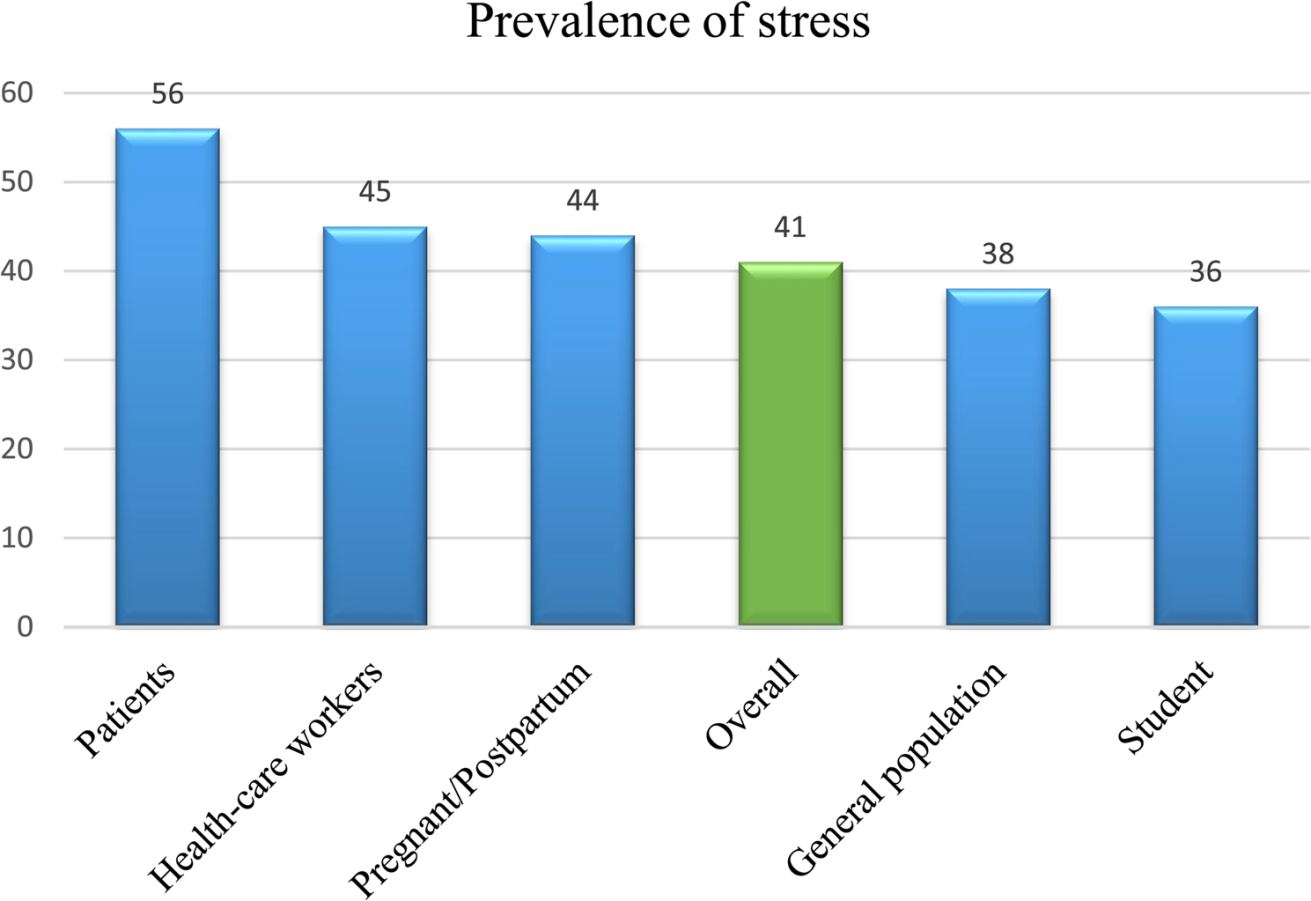
Prevalence of stress according to the groups

**Fig. 4:**
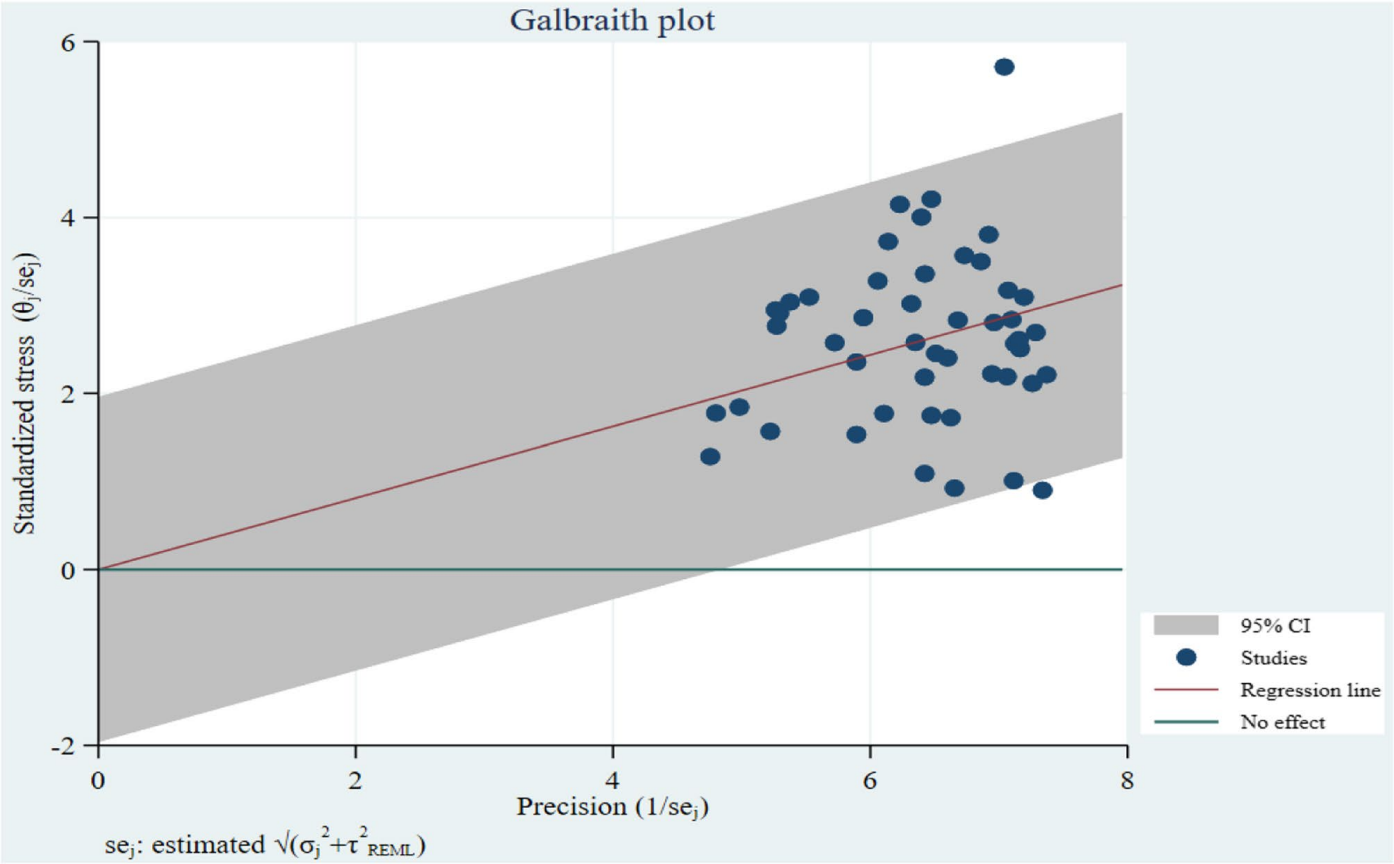
Heterogeneity analysis using Galbraith plot

The pooled prevalence of stress across all studies was 41% (95% CI: 36%–45.0%) (Table 1). Subgroup analysis by population revealed the highest stress prevalence among patients (56% [49%–63%]) and healthcare workers (45% [38%–52%]), followed by other groups (Figs. 2 and 3). Heterogeneity was substantial (*I*² = 93.22%) (Table 1; Fig. 4).

**Table 1:** Detailed information about studies

Variable	Number of Input	Prevalence%[95% CI]	Heterogeneity (I^2^%)	Begg’s and Egger’s test (*P*-value)	Trimfill test (Observed + imputed)
Total	49	41[36–45]	93.22	0.87 and 1	35.6%[30.7–40.4]
Patients	3	56[49–63]	0.01		
Health-care workers	27	45 [38–52]	97.31		
Pregnant/perinatal	3	44[24–65]	55.32		
General population	8	38 [26–49]	81.61		
Students	4	36[26–46]	32.82		
Total in Subgroup	45	44[39–49]	95.45		
Gender	5	35[19–51]	93.17		
Male	2	27[3–50]	85.65		
Female	3	40[18–63]	92.25		
Severity	16	20[12–28]	97.07		
Mild	5	24[6–41]	97.74		
Moderate	4	29[7–50]	96.45		
Severe	7	13[5–21]	90.23		

*CI* Confidence Interval

### Publication bias and sensitivity analysis

**Fig. 5:**
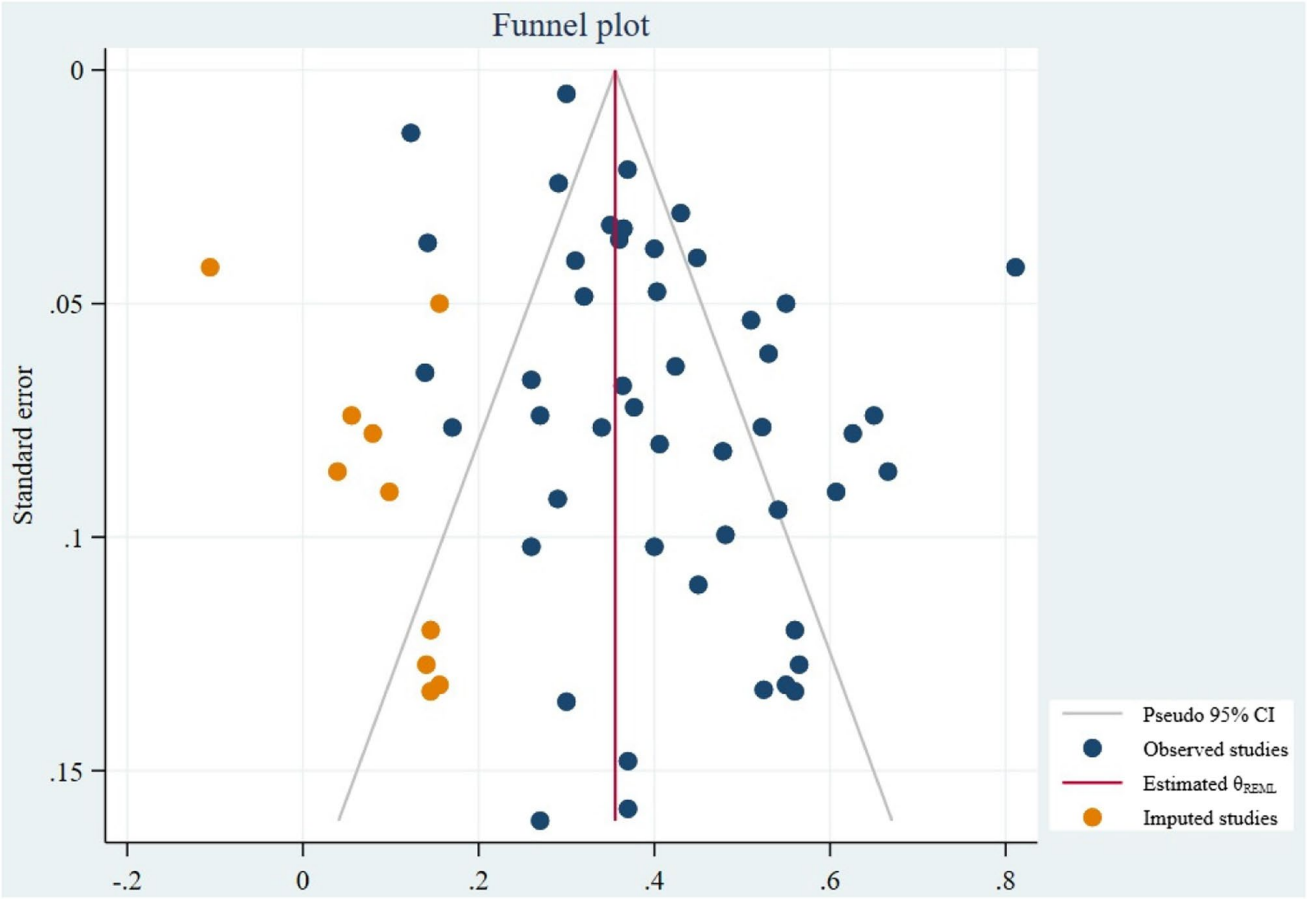
Funnel plot to evaluate the possibility of publication bias

While the funnel plot suggests the potential for publication bias, the statistical tests (Begg’s and Egger’s) do not provide sufficient evidence to confirm the presence of publication bias. The trim-and-fill analysis estimated 10 missing studies; their imputation reduced the overall prevalence to 35.6% (95% CI: 30.7%–40.4%), suggesting a modest overestimation in the initial model (Table 1; Fig. 5). A leave-one-out sensitivity analysis showed the robustness of the pooled prevalence estimate, with no single study having a disproportionate impact on the overall results.

#### Gender

According to the gender variable (Table 1), the estimated overall prevalence of stress was higher in females (40% [95% CI: 18–63]) than in males (27% [95% CI: 3–50]).

#### Severity

In terms of severity (Table 1), the highest percentage was related to moderate, mild, and severe with 29% [95% CI: 7–50], 24% [95% CI: 6–41], and 13% [95% CI: 5–21], respectively.

### Meta-regression

The results of the meta-regression with a random effects model based on the different time periods between the start of COVID-19 and the last date of the search in the articles (week) showed that each past week of Covid-19 increases the prevalence of stress by almost − 0.0000464% [95% CI: −0.0006392, 0.0005464], P-value: 0.878. These results show that “time” is not a significant predictor of an increase in the prevalence of stress.

### Quality assessment

The median quality score was 10 (with a mean of 10.04 out of 11), with 42 studies rated high-quality (9-11) and 3 studies medium-quality (5-8) (Appendix 2).

## Discussion

The prevalence of stress in this study was estimated 41%. According to research studies reviewed by WHO and others showed that the rate of stress during the COVID-19 pandemic varied a lot, with some studies reporting as low as about 8% and others finding rates over 50%, depending on the group of people and the situation [9]. An umbrella review synthesized data from over 70 meta-analyses found that the pooled prevalence of stress among healthcare professionals during the COVID-19 pandemic was approximately 37%, highlighting a significant mental health burden in this populatio [64]. Although, Some studies found no significant changes in psychological distress or stress levels during the COVID-19 pandemic compared to pre-pandemic times, indicating that for certain populations, stress remained stable despite the pandemic [65].

The highest prevalence according to population group was 56% in patients, 45% in health-care workers and 44% in Pregnant/perinatal. The prevalence of stress among patients is usually more than other groups. Nearly half of patients(around 46.6%) diagnosed with COVID-19 experienced severe stress during their illness, according to studies assessing their psychological health [66]. The Research in 2021 on patients discharged from critical care units showed that stress affects 57.8% of patients after leaving critical care units, exceeding the rates of depression (46.5%) and anxiety (53.6%). Variables such as patient age, duration of hospitalization, use of mechanical ventilation, and the specific type of care unit have been shown to significantly influence stress levels among patients [67].

The prevalence of stress in Health-care workers was estimated 45%. Research findings demonstrated that the COVID-19 pandemic led to a substantial rise in stress among healthcare professional. This psychological burden was particularly pronounced among doctors, nursing staff, and those working directly with COVID-19 patients on the frontlines [68, 69]. Studies consistently showed that healthcare workers experienced higher rates of mental health challenges compared to the general public during this period [70]. Various investigations revealed that factors such as fear of transmitting the virus to family members, increased workload and continuous exposure to infected patients contributed significantly to this elevated stress [71, 72].

The prevalence of stress in Pregnant/perinatal was estimated 44%. The numerous systematic reviews have identified common psychological challenges such as fear, anxiety, and depressive symptoms among this population worldwide [73]. Factors contributing to increased stress included younger maternal age, first-time pregnancies, limited social support, unplanned pregnancies, financial difficulties and concerns about contracting or transmitting the virus to the baby [74]. The pandemic also caused some pregnant women to reduce prenatal care visits and increase social isolation, which further exacerbated mental health issues [75]. The findings emphasized the importance of providing strengthened social support, counseling services and specialized mental health care for pregnant and perinatal women during public health emergencies to reduce stress and prevent negative impacts on both mothers and newborns [76].

Moreover, Our results indicated that stress was more prevalent among females (40%) compared to males (27%). This gender disparity aligns with findings from other studies, including a meta-analysis that reported women experienced higher levels of psychological distress than men during the COVID-19 pandemic [77, 78].

In terms of the severity levels of stress, Our analysis indicated that moderate stress was the most frequently reported, affecting 29% of individuals, followed by mild stress at 24% and severe stress at 13%. Similarly, other studies have found that while many people experienced mild symptoms during the COVID-19 pandemic, a significant proportion also faced moderate to severe stress [79, 80].

Analysis of the data indicated that the time elapsed since the start of COVID-19 was not a significant determinant of stress levels. This suggests that, potentially due to the development of effective treatments and vaccines, the population’s vulnerability to the disease decreased and stress levels did not continue to rise indefinitely. The study had several limitations. First, the search was conducted in English, which is common in this study and most of the studies reviewed. Second, the researchers wanted to combine the findings of published meta-analyses so did not exclude the duplicate original studies included in meta-analyses. In addition, the heterogeneity assessment showed that there was a possibility of publication bias. However, the results of the trim and fill test indicated that if such bias occurred, its impact on the study’s results would not be significant. Nevertheless, findings must be interpreted with caution.

## Conclusion

The result showed that during the COVID-19 pandemic, stress was highly prevalent, especially among patients, healthcare workers and pregnant or perinatal individuals. Policy responses should prioritize funding, policy development, advocacy and system improvements to mitigate the pandemic’s mental health impact and enhance resilience across populations when it comes to another pandemic in future. Policymakers should prioritize high-risk populations during pandemics by integrating early-stage mental health interventions into public health strategies. Training programs focused on prevention, timely detection of stress symptoms, and facilitating prompt access to treatment can significantly mitigate stress prevalence, promote earlier help-seeking behavior, and reduce the overall psychological burden on both individuals and society.

## Data Availability

Data is provided within the manuscript or supplementary information files.

## Appendix 1

**Table 2:** Sample of search strategy for PubMed

494,687	(Stress [Title/Abstract]) OR (Mental Disorders [Title])) OR (Psychological impact[Title])) OR (Psychological consequences[Title])) OR (Psychological Disorders [Title])) OR (Mental health [Title])) OR (Psychological[Title])
369,143	(Covid[Title]) OR (2019 novel coronavirus[Title])) OR (COVID19[Title])) OR (COVID-19[Title])) OR (COVID 2019[Title])) OR (2019-novel CoV[Title])) OR (SARS-cov-2[Title])) OR (SARS-CoV2[Title])) OR (SARSCoV2[Title])) OR (SARSCoV-2[Title])) OR (severe acute respiratory syndrome coronavirus 2[Title])) OR (2019-ncov[Title])) OR (coronavirus disease 2019[Title])) OR (coronavirus disease-19[Title])) OR (2019ncov[Title])) OR (SARS coronavirus2[Title])) OR (severe acute respiratory syndrome coronavirus 2[Title])) OR (COVID-19[Title])
479,679	(Meta analysis[Title/Abstract]) OR (Meta-analysis[Title/Abstract]) OR (Systematic Review[Title/Abstract])
3,697	#1 AND #2 AND #3

## Appendix 2

**Table 3:** Summary of selected studies

First author, Publication Year	Sample	Sample Size	Last date of search	Prevalence (95%CI)		Severity %Prevalence[CI] (Sample Size)	Quality score
				%Prevalence[CI]	Subgroup %Prevalence[CI] (Sample Size)		
Luo M, 2020 [19]	Medical staff and general public	162,639	25/5/2020	40[20-60]			8
Salari N, 2020, [20]	Front-line healthcare workers	3819	../6/2020	45[24.3–67.5]	-Hospital staf (non-physicians and nurses): 36.4[18.3-59.5] (3551) -Physicians: 93.7[90-96] (268)		9
Batra K, 2020 [21]	Healthcare Workers	16235	27/7/2020	40.3[31.4–50.0]		-Mild: 25.8[16.8–37.6] -Moderate: 52.3 [38.7–65.5] -Severe: 18.9[11.9–28.9]	11
Krishnamoorthy Y, 2020 [22]	General population, healthcare workers and COVID-19 patients	8140	22/4/2020	Stress:34[20-50]	-General population: 36[5-75] -HCW:33[19-50]		11
Singh RK, 2021 [23]	Health care workers and general population	3016	10/10/2020	60.7[42.3–77.7]	-HCW: 65.1[48.2%–80.3] -General population: 51.7[05.3–96.2]		7
Zhao Y-J, 2021 [24]	General populations and health professionals	6531	25/5/2020	14.2[8.4 - 22.9]			11
Marvaldi M, 2021 [25]	Healthcare workers	2845	8/10/2020	56.5[30.6–80.5]			8
Sousa GMd, 2021 [26]	General public (GP) and health care workers	1,074,438	2/3/2021	36[29.31–43.54]			9
Mahmud S, 2021 [27]	health professionals	82783	30/3/2021	44.86[36.98–52.74]			10
Al Maqbali M, 2021 [28]	nurses	27034	26/10/2020	43[37–49]			11
Bareeqa SB, 2021 [29]	Chinese people	18439	../4/2020	48.1[28.7–67.7]			10
Dong F, 2021 [30]	health care workers	13788	7/10/2020	29.1[24.3–33.8]			11
El-Qushayri AE, 2021 [31]	healthcare workers	1911	15/1/2021	66.6[47.6-81.3]		-Severe: 14.3[10.0-20.1] -Mild: 11.5[9.5-13.9] -Very severe: 9.3[3.2-24.0]	10
Dutta A, 2021 [32]	Health-care workers	27238	15/8/20	37.7[24.0–52.3]			10
Varghese A, 2021[33]	Nurses	4204	5/10/20	40.6[25.4-56.8]		-Mild:2.9 [0.2-8] (421) -Moderate:2.9 [0.2-8] (421) -Severe:2.3 [0-13.2] (421) -Extreme severe:0.8 [0-5.3] (421)	11
Demissie DB, 2021 [34]	Women who are pregnant and/or lactating	1765	30/9/20	56 [30.07-82.22]			10
Mulyadi M, 2021 [35]	Nursing students	817	29/6/21	30[10-63]			10
Nochaiwong S,2021 [36]	General population	110849	16/6/20	36.5[30-43.3]			11
Yan H, 2021 [37]	Medical Staff	25,343	19/4/2020	56[32–79]			11
Ching SM, 2021 [38]	healthcare providers	34010	15/3/2021	36.4[23.2–49.7]	-Females:48.1[31.6–64.5] -Males:40.4[22.8–57.9]		11
Halemani K,2021 [39]	Health-care workers	9060	31/4/2021		-Nurse : 37[0.8-0.66] -doctor: 37[0.6-0.68]		10
Aymerich C, 2022 [40]	Health worker’s	48042	1/3/2020	40[32–47]			9
Alzahrani F, 2022 [41]	General population	4478	17/8/2020	29[11-47]			10
Ghahramani S, 2022 [42]	Healthcare Workers	53463	20/2/2022	27[6–69]			11
Blasco-Belled A, 2022 [43]	general population and healthcare workers	880,352	6/12/2020		-General population:35[0.28-0.41] -Healthcare workers:32[0.23-0.42]		9
Arora T, 2022 [44]	healthcare workers and general population	97173	../4/2020	27[14–43]			10
Gao S, 2022 [45]	Postpartum women	1429	16/6/2021	55[27.9–79.5]			11
Cheung T, 2022 [46]	Healthcare Workers, general population and affected individuals	27,325	1/6/20	54.1[35.7–72.6]			10
Huang G, 2022 [47]	first responders for medical emergencies	265		17[4-34] (265)		-Mild: 58[38-77] (26) -Moderate: 22[5-44] (26) -Severe: 19[5-37] (26)	10
Ma K, 2022 [48]	Teachers	256,896	../7/2021	62.6[46.1–76.6]			10
Fang Y, 2022 [49]	student	855564	10/3/2022	31[23-39]	-Male: 0.16 [0.12-0.21] -Female: 0.19 [0.12-0.28] -Medical: 0.28 [0.18-0.40] -Non-medical: 0.41 [0.29-0.54]		11
Tong J, 2023 [50]	Frontline healthcare workers	5976	31/12/2021				10
Aldhamin RA, 2023 [51]	Healthcare workers	16071	../4/2021			-Moderate to severe stress (PSS-10): 81.12[72.15-88.70] (1871) -Moderate to severe stress s (DASS-21): 12.29[9.77-15.05] (602)	9
Hasen AA, 2023 [52]	Healthcare professionals	3815	../6/2022	51[0.41-0.62]			10
Asfaw Hasen A,2024 [53]	healthcare professionals	5461	31/3/2023	57.27[42.28-72.25]			10
Al Maqbali M,2024 [54]	Health-care workers	843621	15/1/2024	37[32.87-41.22]	-Nurse: 42.4[30.4-52.27]		10
Aljuwaiser S,2024 [55]	medical students and junior doctors	16277	15/5/2022	29.6 - 49.9			9
BadilloSánchez N,2024 [56]	Nursing students	8107	31/1/2024	52.46[26-78]			10
HuangI J,2024 [57]	Nurses,doctors and other medical staff	5776	11/11/2022	30[29-31]			10
Iranifard E,2024 [58]	Infertile patients	5901	31/12/2022	55[45.4-65]	Female 0.554[0.454-0.650]		11
Jalali A,2024 [59]	the elderly population	2306	31/12/2023	13.90[5.5-30.9]			10
Panahi P,2024 [60]	patient with epilepsy	611	3/31/2023	48.7[32.8-64.8]			11
Safia A,2024 [61]	allergic rhinitis patients	18269265	25/10.2023	65[14-36]		-Low: 28[10-45] -Moderate: 39[32-45] -High: 33[21-45]	10
Abdul Aziz H,2025 [62]	Pregnant Women	61719	31/1/2023	26[9-49]			10
Bidhendi-Yarandi R,2025 [63]	student,General population and health-care workers	11609386	31/8/2024	26[13-29]			11
